# Testosterone and Quality of Life in Transgender and Gender-Diverse Adults Seeking Masculinization: A Secondary Analysis of a Randomized Clinical Trial

**DOI:** 10.1001/jamanetworkopen.2024.43466

**Published:** 2024-10-25

**Authors:** Brendan J. Nolan, Sav Zwickl, Peter Locke, Ada S. Cheung

**Affiliations:** 1 Trans Health Research Group, Department of Medicine (Austin Health), University of Melbourne, Heidelberg, Victoria, Australia; 2 Equinox Gender Diverse Clinic, Thorne Harbour Health, Abbotsford, Victoria, Australia; 3 Department of Endocrinology, Austin Health, Heidelberg, Victoria, Australia

## Abstract

This secondary analysis of a randomized clinical trial evaluates the quality of life outcomes of immediate testosterone commencement compared with a 3-month waiting period.

## Introduction

Testosterone treatment is a necessary component of gender affirmation for some transgender and gender-diverse adults seeking masculinization. We have previously demonstrated^1^ that early access to testosterone therapy, compared with commencement following a standard care waiting list of 3 months, reduces gender dysphoria, depression, and suicidality in transgender and gender-diverse adults. Observational studies^2,3^ have demonstrated associations between testosterone commencement and improvements in quality of life; however, no randomized clinical trial has been performed that we know of. We aimed to examine the outcomes of early access to testosterone therapy, compared with no treatment, on quality of life and hypothesized that early access to testosterone therapy would improve quality of life.

## Methods

In this prespecified secondary analysis of a randomized clinical trial, we recruited transgender and gender-diverse adults seeking initiation of testosterone as previously described.^1^ Participants provided written informed consent, and the trial was approved by the human research ethics committee at Austin Health and registered with the Australian New Zealand clinical trials registry (ANZCTR 12621000716864). This study followed the Consolidated Standards of Reporting Trials (CONSORT) reporting guideline. The trial protocol is available in Supplement 1, and the CONSORT flow diagram is shown as eFigure in Supplement 2.

Participants were randomized to immediate testosterone commencement (intervention group) within 1 week of the first study visit or no treatment (commencement of testosterone following a standard care waiting list of 3 months). Participants completed the EuroQol (EQ-5D-5L) quality of life questionnaire at baseline and 3 months.^4^ The descriptive system comprises 5 dimensions: mobility, self-care, usual activities, pain and/or discomfort, and anxiety and/or depression, where each dimension has 5 potential responses (from no to extreme problems). Utility scores were generated using the UK general population-based value set, where values range from −0.285 to 1, and values less than 0 indicate a state worse than death.^5^ The EQ-VAS is a visual analogue scale that enables participants to assign a value to their overall health rating using a visual analogue scale from 0 (“The worst health you can imagine”) to 100 (“The best health you can imagine”).

Analysis of covariance was used to estimate the mean difference and corresponding 95% CI between the intervention group and the standard care group, adjusted for the corresponding measure at baseline.^6^ A 2-tailed significance level of *P* < .05 was used. Statistical analyses were performed using STATA version 17.0 (StataCorp LLC) from July to September 2023.

## Results

Of 64 transgender and gender-diverse adults enrolled in the study, 62 completed the 3-month follow-up (31 in the intervention and standard care arms). Baseline characteristics have been previously published and are reproduced in Table 1.^1^ Compared with controls, in individuals receiving testosterone, there was a clinically significant improvement in EQ-VAS over 3 months of follow-up (mean difference, 11.6 points; 95% CI, 4.9 to 18.3 points; *P* = .02) (Table 2). The between-group change in EQ-5D-5L utility index was not significant (mean difference, 0.07 points; 95% CI, −0.07 to 0.21 points; *P* = .06). In a sensitivity analysis, there were no between-group differences in the 5 dimensions of EQ-5D-5L (Table 2).

**Table 1.** Baseline Characteristics

Characteristic	Participants, No. (%)	
	Intervention (n = 31)	Standard care (n = 31)
Age, median (IQR), y	23 (20-28)	22 (20-27)
Gender identity		
Binary	17 (55)	15 (48)
Nonbinary	14 (45)	16 (52)
Comorbidities		
Depression	23 (74)	18 (58)
Anxiety	20 (65)	20 (65)
ASD	8 (26)	4 (13)
ADHD	8 (26)	6 (19)
Residence		
Metropolitan	23 (74)	25 (81)
Rural/remote	8 (26)	6 (19)
Employment status		
Employed/student	23 (74)	26 (84)
Unemployed	8 (26)	5 (16)
Total testosterone, mean (SD), ng/dL	37.5 (20.2)	25.9 (14.4)

Abbreviations: ADHD, attention deficit hyperactivity disorder; ASD, autism spectrum disorder.

SI conversion factor: To convert testosterone to nmol/L, multiply by 0.0347.

**Table 2.** Quality of Life Measures in Individuals Receiving Immediate Commencement Testosterone Compared With Those Receiving Standard Care

Parameter	Measure, Mean (SD)		Mean difference (95% CI)^a^	*P* value
	Intervention (n = 31)	Standard care (n = 31)		
EQ-5D-5L utility index				
0 mo	0.70 (0.21)	0.74 (0.17)	0.07 (−0.07 to 0.21)	.06
3 mo	0.79 (0.16)	0.74 (0.19)		
EQ-5D-5L VAS				
0 mo	56.6 (19.8)	57.0 (21.7)	11.6 (4.9 to 18.3)	.02
3 mo	69.1 (20.6)	57.6 (21.9)		
EQ-5D-5L dimensions				
Mobility				
0 mo	1.35 (0.75)	1.19 (0.48)	0.13 (−0.08 to 0.34)	.21
3 mo	1.23 (0.62)	1.26 (0.51)		
Self-care				
0 mo	1.77 (0.92)	1.68 (0.79)	0.34 (−0.01 to 0.68)	.06
3 mo	1.45 (0.62)	1.74 (0.93)		
Usual activities				
0 mo	2.13 (1.02)	2.26 (0.89)	0.13 (−0.35 to 0.60)	.54
3 mo	1.94 (1.12)	2.13 (0.96)		
Pain/discomfort				
0 mo	1.87 (0.96)	1.65 (0.88)	0.35 (−0.06 to 0.76)	.09
3 mo	1.61 (0.76)	1.87 (0.99)		
Anxiety/depression				
0 mo	3.00 (0.89)	2.81 (0.95)	0.40 (−0.06 to 0.86)	.08
3 mo	2.35 (1.08)	2.65 (1.02)		

Abbreviations: EQ-5D-5L, EuroQol; VAS, visual analogue scale.

^a^ The mean difference and 95% CI refer to the between-group difference over 3 months adjusted for the corresponding measure at baseline.

## Discussion

In this secondary analysis of a randomized clinical trial of transgender and gender-diverse adults seeking initiation of testosterone, there was a clinically meaningful improvement in self-rated health via the EQ-VAS, but the change in EQ-5D-5L utility index was not significant. Study limitations include the short follow-up period, open-label study design, and that EQ-5D-5L has not been validated in the transgender and gender-diverse population. Some of the EQ-5D-5L dimensions, such as mobility or pain, would not necessarily be expected to improve with testosterone. In contrast with our previous findings,^1^ we also did not find a difference in the anxiety and/or depression dimension of EQ-5D-5L, which could indicate a lack of sensitivity of the outcome measure or insufficient sample size.

Our findings support the use of testosterone to improve self-reported health. Further research is needed to determine the optimal quality of life measure in the transgender and gender-diverse population.

## Notes

Supplement 1.
Trial Protocol

Supplement 2. **eFigure.** Study Subjects and Flow of Participants

Supplement 3.
Data Sharing Statement
