# Supplementary material for: Testosterone and Quality of Life in Transgender and Gender-Diverse Adults Seeking Masculinization: A Secondary Analysis of a Randomized Clinical Trial
Source: JAMA Netw Open. 2024 Oct 25;7(10):e2443466. doi: 10.1001/jamanetworkopen.2024.43466 (PMC11581477; doi:10.1001/jamanetworkopen.2024.43466)
Supplement: Supplement 2. — eFigure. Study Subjects and Flow of Participants [file jamanetwopen-e2443466-s002.pdf]

Supplemental Online Content

Nolan BJ, Zwickl S, Locke P, Cheung AS. Testosterone and quality of life in transgender and gender-diverse adults seeking masculinization. *JAMA Netw Open*. 2024;7(10):e2443466. doi:10.1001/jamanetworkopen.2024.43466

eFigure. Study Subjects and Flow of Participants

This supplemental material has been provided by the authors to give readers additional information about their work.

© 2024 Nolan BJ et al. *JAMA Network Open.*

eFigure. Study Subjects and Flow of Participants

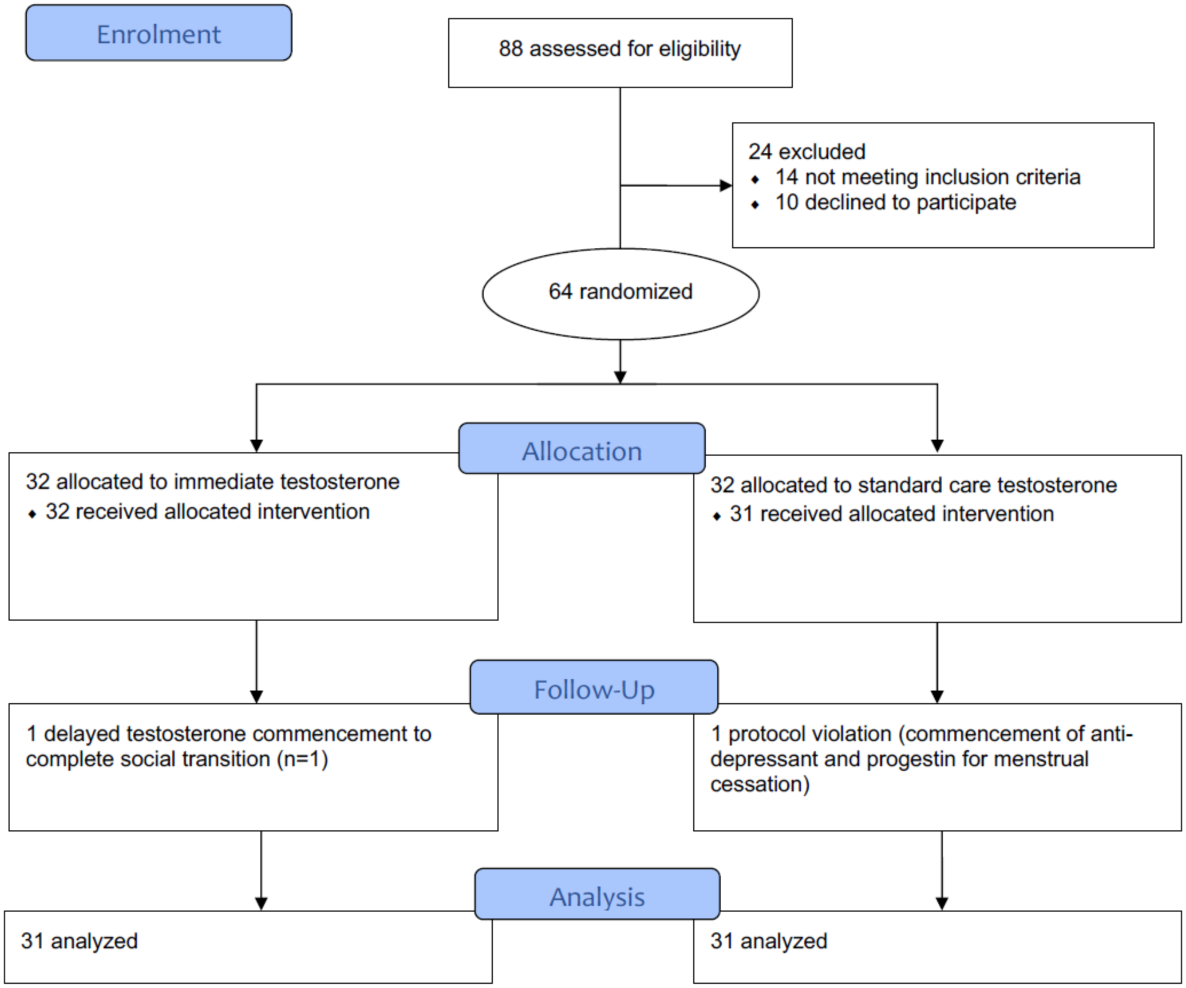

© 2024 Nolan BJ et al. *JAMA Network Open.*
