# Supplementary material for: Testosterone and Quality of Life in Transgender and Gender-Diverse Adults Seeking Masculinization: A Secondary Analysis of a Randomized Clinical Trial
Source: JAMA Netw Open. 2024 Oct 25;7(10):e2443466. doi: 10.1001/jamanetworkopen.2024.43466 (PMC11581477; doi:10.1001/jamanetworkopen.2024.43466)
Supplement: Supplement 3. — Data Sharing Statement [file jamanetwopen-e2443466-s003.pdf]

Data Sharing Statement

Nolan. Testosterone and Quality of Life in Transgender and Gender-Diverse Adults Seeking Masculinization. *JAMA Netw Open*. Published October 25, 2024.
doi:10.1001/jamanetworkopen.2024.43466

Data
Additional Information: Australian New Zealand Clinical Trials Registry
https://www.anzctr.org.au/Trial/Registration/TrialReview.aspx?id=381836&isReview=true
ACTRN12621000716864
Data available: Yes
Data types: Deidentified participant data
How to access data: nolan.b@unimelb.edu.au
When available: With publication

Supporting Documents
Document types: None

Additional Information
Who can access the data: Researchers whose proposed use of the data has been approved
Types of analyses: For a specified purpose
Mechanisms of data availability: With a signed data access agreement
